# Remote exercise assessment in pulmonary hypertension

**DOI:** 10.1097/MCP.0000000000001277

**Published:** 2026-05-07

**Authors:** Paula Appenzeller, Daniel Jeffery, Mark Toshner

**Affiliations:** a Royal Papworth Hospital NHS Foundation Trust; b Victor Phillip Dahdaleh Heart & Lung Research Institute, University of Cambridge, Cambridge, UK

**Keywords:** actigraphy, digital health technology, exercise assessment, six-minute walk test, pulmonary hypertension

## Abstract

**Purpose of review:**

In pulmonary arterial hypertension (PAH), assessment of exercise provides important prognostic information. Recently, digital alternatives to traditional outcome measures, including digital versions of the six-minute walk test (6MWT), have been proposed. This review discusses existing methods of remote exercise assessment in PAH.

**Recent findings:**

Summary metrics from actigraphy (e.g. daily steps) show promise on a population level but show high variability (e.g. related to seasonality) that may obscure clinically important changes. Conversely, digital structured exercise tests (6MWT) have proven safe, accurate compared to gold-standard tests and well-accepted by patients. Implementation and underlying algorithms vary, depending on whether tests are performed indoors or outdoors, along fixed or free courses, using accelerometery or GPS, and are delivered through app-only or app-and-wearable platforms. Integration of physiological data from wearables enhance digital 6MWT performance and hold promise as longitudinal endpoints. Key challenges include continued patient adherence and rigorous pre-processing of the raw data to ensure sustained data quality.

**Summary:**

Future studies are needed to demonstrate the ability of the digital 6MWT or alternative exercise measures to reflect disease severity, show sensitivity to change and establish minimal clinically important differences for them to be implemented in clinical care for longitudinal monitoring.

## INTRODUCTION

Exercise assessment plays an important role in pulmonary arterial hypertension (PAH), a rare condition characterized by adverse remodelling of the pulmonary arteries leading to increased pulmonary vascular resistance and presenting with dyspnoea and exercise limitation [1]. Current treatment aims to achieve and maintain a low mortality risk profile, defined by multiparametric risk assessments, which includes longitudinal assessment of structured exercise tests such as the six-minute walk test (6MWT) [1,2]. In PAH, the 6-minute walk distance (6MWD) associates with functional capacity, clinical deterioration, and long-term survival, thus it is a widely recognized endpoint for trials [1–3] as well as being incorporated into multiple risk scores [4–6].

PAH is rare, and in many health systems, care is centralized. In the UK, for example, there are only eight specialist centres, which are also the focus of almost all research activity, despite patients living throughout the country, which results in patients travelling significant distances to access specialist care [7]. This places considerable burden on these patients. Accordingly, clinical assessments which include the 6MWT, are often limited to once or twice a year. Digital technologies could address this limitation by providing means for more frequent assessment. Globally, 93.4% of patients living with pulmonary hypertension report owning a smartphone [8], and there is increasing interest from clinical services and research regulators in developing novel approaches to patient care utilizing digital technologies [9,10]. The National Health Service (NHS) UK published its 10-year plan outlining the expectation that patients will have routine access to wearable technology as part of their healthcare [11], enabling care to shift from hospital to community and from analogue to digital. Recently, regulators approved remotely collected activity measures as the primary endpoint in two clinical trials [12,13]. Remote data collection allows patients to perform exercise tests in the community at their convenience. Over recent years, several research centres developed a digital equivalent for the 6MWT, and continuous actigraphy is becoming more popular. However, methods differ between centres, and currently, there is little consensus between researchers as to the optimal solution, and some key challenges including continued patient adherence persist. 

**Box 1:**
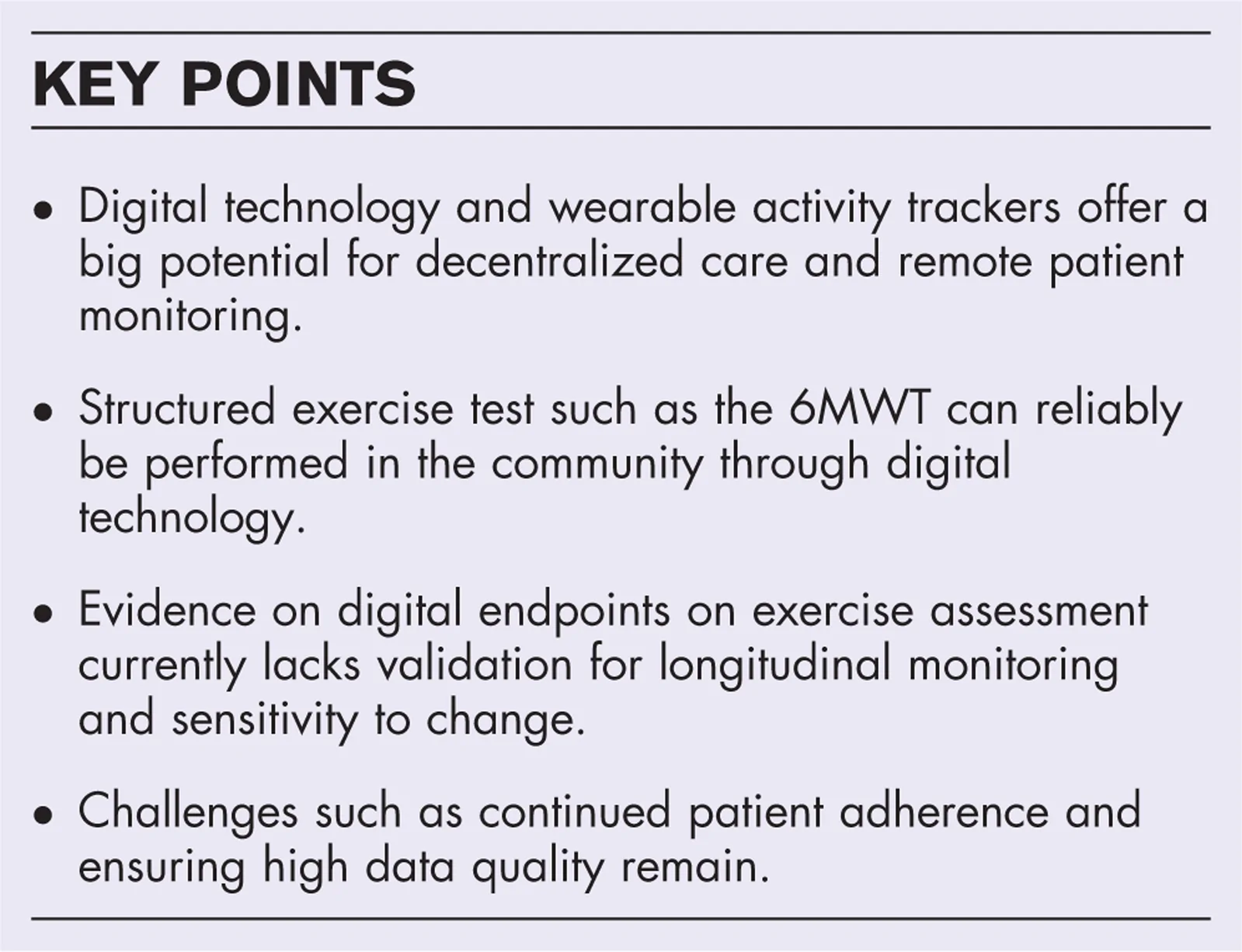
no caption available

## REVISITING TRADITIONAL ACTIGRAPHY

Actigraphy, the quantification of physical activity, is a well-established monitoring parameter in PAH and smartphones or wearable sensors offer intriguing opportunities to collect continuous actigraphy data [10]. Early studies demonstrated that, in PAH, all levels of physical activity measured by wrist-worn accelerometers were reduced with increased sedentary time compared to healthy controls [14–19]. In a recent structured review, Robertson *et al*. noted, that while some measures of physical activity were shown to correlate with clinical endpoints, they correlated poorly with haemodynamic variables [20^▪^]. Additionally, more recent studies suggest that conventional accelerometery-derived metrics such as average daily steps may lack sensitivity to detect treatment-related changes [15,21–23]. Passively collected data have also been shown to demonstrate seasonal variations independent of haemodynamic parameters or structured exercise tests [24^▪^]. This suggests that conventional summary metrics may be influenced by external factors unrelated to disease severity and thus may not be specific enough to differentiate clinically relevant changes in long-term monitoring.

In contrast, data from MyHeartCounts demonstrated that large-scale clustering of activity patterns on a population level shows significant differences. In a population of almost 50,000 participants, a pattern of lower overall activity but more frequent transitions between active and inactive states associated with self-reported cardiovascular disease [25]. While actigraphy may help detect overall clinical patterns, derived metrics or structured exercise tests seem to show more promise for disease-specific and long-term monitoring.

## DIGITAL SIX-MINUTE WALK TEST

Several groups have aimed to recreate the in-clinic 6MWT using digital technology. LaPatra *et al*. demonstrated that the 6MWT can be replicated in the community with good correlation between remote and in-clinic tests [26]. Their approach requires another person to be present to count distance, a flat surface, which can replicate the 6MWT corridor and a member of the research team to virtually supervise. As such, it is still relatively resource intensive, which limits the potential broader scale utility. There is increasing recognition that mobile technology can be harnessed to gain longitudinal, remote assessments of exercise capacity using the 6MWT without the same level of input from clinical services. Several different approaches have been developed, which can be divided into categories; app-only vs. app-and-wearable platforms; using accelerometery data vs. Global Positining System (GPS) to record distance walked, performing the test indoor vs. outdoor; and walking back and forth a predefined course vs. choosing a free course. Table 1 gives an overview of pulmonary hypertension-specific studies comparing the correlations to in-clinic tests and methodology.

**Table 1:**
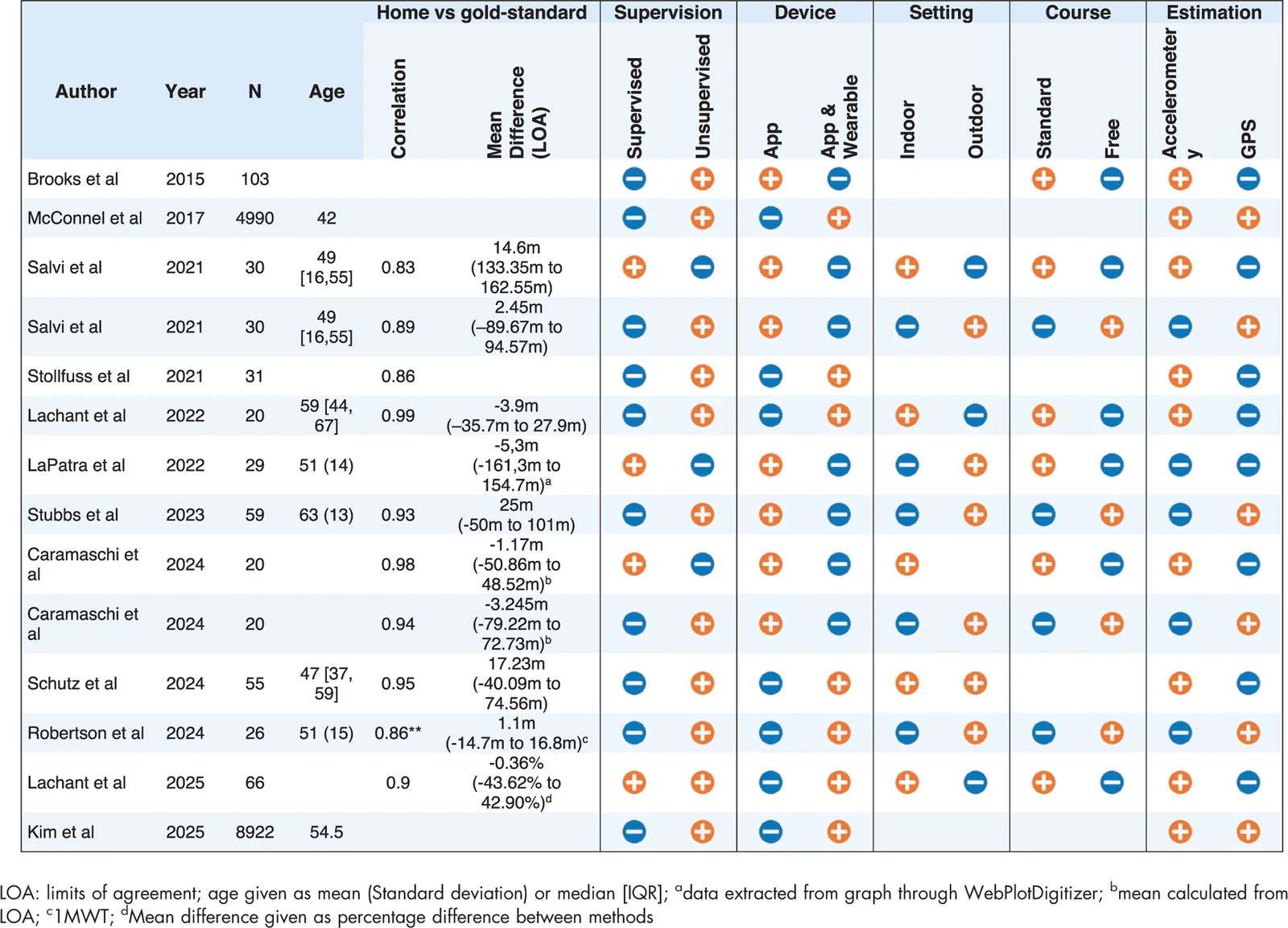
Comparing different methodologies of digital six-minute walk tests and their agreement to the gold-standard in-clinic six-minute walk test across the last decade

### App-only platforms

This approach is attractive, as it takes advantage of the wide availability of smartphones and the proliferation of health and exercise-focused apps. The largest app-based 6MWT platform is MyHeartCounts [25,27], which has collected over 30 000 digital 6MWT from over 9000 individual participants, demonstrating the wide-spread uptake throughout the world. While not pulmonary hypertension-specific, this study includes pulmonary hypertension patients and demonstrated that a digital 6MWT can be reliably performed in the community by healthy volunteers and patients living with cardiovascular disease. Remote monitoring was sensitive enough to detect differences between conditions and change over time [27]. App-based platforms have been used in a range of studies and were shown to be safe and in good agreement with in-person 6MWT [28–31,32^▪^] (Table 1). They are simple and comparatively easy to use, which widens accessibility. The data they provide are from either accelerometery sensors embedded within the phone or from GPS data and therefore cannot provide physiological data such as heart rate (HR), which can provide invaluable additional context. These can only be obtained using additional sensors.

### Additional wearable sensors

To address the lack of physiological data, many groups have paired app-based data collection with a wearable device such as a smartwatch [33,34^▪^,^35^] or chest-based ECG sensor [36,37^▪▪^]. Using these devices adds a layer of complexity for participants, as they have to connect and start a second device, with associated additional risk of technical problems. In general, patients are more likely to embed technology in everyday life when it requires minimal additional effort.

A well-recognized limitation of the 6MWT is that it is a motivation-dependent test. This introduces an element of variation [38,39], which could be assessed through measurement of ‘cardiac effort’ [36], the number of heart beats divided by the 6MWD. Thus, incorporating HR metrics measured by wearable sensors into the 6MWT allows some account to be taken of variation in effort. This is conceptually interesting, and Lachant *et al*. were able to show that, while there was variation in the 6MWD between in-clinic and home-based tests, cardiac effort was consistent [36], suggesting that measuring effort is crucial for interpretation of the 6MWD. However, heart rate may be affected by rate-limiting medications such as beta-blockers or calcium channel blockers, which are common in cardiovascular disease, limiting the applicability of cardiac effort in these patients. The incorporation of wearable devices into remote exercise testing offers significant, unexplored possibilities. Using the parameters outlined above either alongside the 6MWD or as passive surrogates of exercise capacity offers new potential endpoints and may ultimately negate the need for regular structured tests all together.

### Accelerometery vs. global positioning system

Another key difference in approach for the remote 6MWT is the method of assessing 6MWD. Many smartphones are now equipped with satellite navigation (GPS, Glonass, Galileo, GNSS) allowing for detailed tracking of distance from both phones and wearables. Digital 6MWTs harnessing this technology [28,29,32^▪^,34^▪^], have been shown to be replicable and well correlated with in-person tests (Table 1). GPS-based tests must be performed outside which has multiple advantages such as allowing participants to walk unhindered. However, outdoor tests expose patients to seasonal and weather variability, and depending on surroundings, device connectivity to satellite systems may be delayed, both of which present barriers to patient adherence, especially in PAH, where many patients have co-existing connective tissue disease. It should also be noted that GPS quality varies across devices, which may impact data quality. Caramaschi *et al*. [32^▪^] demonstrated that, if the GPS quality is low, step count-based methods are more accurate, improving the mean error of measured 6MWD from 93.8 to 22.5 m.

On the other hand, accelerometers allow the test to be performed indoors, as they are not reliant on connecting to satellites. This assumes that patients have sufficient indoor space, which is rarely the case. There have been efforts to shorten the course to 10 m [37^▪▪^] but even this will not be possible for the majority of patients globally. When performed outside, accelerometery-based platforms can simplify the process, as all the technology required is usually embedded in one device. Often 6MWD is calculated from algorithm-derived estimations of stride lengths (based on sex, height, and sometimes calibrated against GPS) and step count [28,31,32^▪^,^35^,^36^,^37^^▪▪^]. Step count can be inaccurate, especially when using wrist-based devices, as sensors are susceptible to arm position and swing (i.e. if patients hold their hands still while walking, the watch may not accurately detect steps [36,40]). Therefore, clear instructions are critical when implementing digital 6MWT to minimize the risk of error being introduced.

### Indoor vs. outdoor and fixed vs. free course

The choice of algorithm or technology is inherently linked to the setting of the test. Many platforms have suggested performing the test along a fixed indoor course [29,32^▪^,^33^], which allows for a more controlled and replicable environment, although it requires access to a straight indoor space. As performing tests indoors prevents the use of GPS, most algorithms detect U-turns from raw accelerometery data and multiply these with the known length of the path [28,32^▪^].

Performing the test outdoors has the obvious advantage of allowing patients significantly more choice of setting and more space. Walking along an unrestricted course minimises turns and so reduces the risk of step-count being miscounted or GPS signals being inaccurate [40]. However, it does introduce environmental factors. which can increase variability, such as weather, traffic, elevation changes, and perceived vulnerability in patients, caused by feeling observed [30,41^▪^]. And notably, choosing a standard fixed course for a GPS-based algorithm leads to significant errors [40]. It follows that the choice of algorithm is influenced by the setting and methodological approach of the test and that adherence to the intended setting is essential to minimize error.

## FUTURE PERSPECTIVES

Simply replicating clinic-based testing does not fully acknowledge the capabilities of wearable technology, and there is a question whether it is necessary to do so, or if alternative endpoints could provide equally meaningful outcomes, if not more so. A digital 1-MWT based on a free outdoor course using GPS data was shown to be equally valid as the digital 6MWT in PAH, with patients showing clear preference for the shorter walk [34^▪^]. Using a shorter test duration also greatly simplifies exercise testing in real-world settings. Wearables additionally offer the possibility to integrate classic endpoints with simultaneously measured physiological parameters, such as cardiac effort [36,37^▪▪^] or heart rate recovery [33], which help to contextualize findings.

Looking beyond structured exercise testing, peak 5-min steps, a measure passively derived from granular accelerometery data and averaged over time, was shown to be sensitive to change after initiation of treatment [42^▪^,^43^] across two trials, while traditional actigraphy data was not. Also, a passive 6MWT derived from the average maximal steps within 6 min has recently been explored as a measure of exercise capacity in both PAH [44] and patients with heart failure [45], where it was shown to correlate with both 6MWD and peak oxygen uptake in patients over 65 years of age.

In a qualitative interview study, pulmonary hypertension patients identified subjective alternatives to active testing including the ability to walk uphill and the duration of continuous walking [41^▪^]. In line with this, a model for early detection of pulmonary hypertension through longitudinal activity monitoring, derived from the MyHeartCounts platform, incorporates flights climbed alongside other variables derived from smartwatches [46]. While new measures beyond structured exercise tests show promise, it is currently unclear whether they could replace active exercise testing or whether they represent daily functioning, more akin to the WHO Functional Class. It is important to further examine this in clinical trials to allow appropriate clinical interpretation.

Finally, artificial intelligence offers vast and, as yet, unexplored potential to facilitate the deployment of remote exercise testing at scale. These potentially include machine learning models which take into account environmental and conditional circumstances to improve the standardization of remotely delivered 6MWTs, or improved data quality control to allow deployment at scale. Artificial intelligence models could potentially integrate high-dimensional activity and physiological data from wearables to identify early markers of deterioration and alert patients and services earlier than would have been otherwise possible. Of course, this technology is still largely in its infancy, and further work is needed before these models are ready to bring into the clinic.

## CHALLENGES WITH REMOTE EXERCISE ASSESSMENTS

While there is a great interest in digital solutions on the patients’ side, with 80% of global patients willing to complete digital walk tests [8], continued adherence to digital solutions remains a major challenge, with trials reporting a discontinuation rate of 15–48% [29,30]. Interactions with the study team or automated alerts in form of text messages [30,47] could help mitigate this problem in clinical trials, but it is questionable if this approach is feasible for clinical implementation in already stretched clinical services.

Patient adherence is directly linked to the frequency of remote assessment. Currently, the optimal duration and frequency of monitoring required to generate stable and meaningful endpoints remains unknown, and there is little evidence on the topic. Longitudinal data provides a great advantage over single measurements, as serial and averaged data can reduce noise produced by high inter-test variability, improving the performance of digitally derived endpoints [33] and thus offering an advantage over traditional in-clinic tests. It is, therefore, necessary to find a balance between optimizing patient's preferences and ensuring data quality.

The latter is particularly challenging given the high-dimensional data derived from digital sensors. Unlike traditional clinical endpoints, digital metrics are more vulnerable to technical artefacts. Rigorous statistical frameworks are needed to ensure their validity. For example, Caramaschi *et al*. [32^▪^] significantly reduced measurement errors when using step-count-based algorithms compared to GPS-based algorithms, when the GPS signal was of low quality. This underlines the potential importance of preprocessing of the data and algorithms that can handle signal-to-noise appropriately. Ensuring data quality and minimal signal errors is also important to determine a minimal clinically important difference (MCID) for digital exercise tests. This is the minimal change between tests, which is felt to be reflective of clinical change relevant to the patient. The MCID for the in-person test is agreed to be 33–39 m [48], but current measurement errors for digital tests and their 95% confidence intervals approach this range (Table 1).

Another area that requires consideration is data missingness. It is important to understand patterns of missing data and develop frameworks to account for this [49]. For example, patients may be less likely to wear their wearable device or perform an exercise test if they are unwell. Consequently, deterioration may not be detected. This phenomenon has not yet been extensively explored but is critical to maximize the utility of these technologies.

## CONCLUSION

Over recent years, technical innovations have transformed exercise assessment in pulmonary hypertension. Digital solutions offer considerable promise for tracking disease progression and treatment response over time. Across multiple different methodologies, it has been shown that in patients with pulmonary hypertension, the digital 6MWT is safe, easy to use and highly accepted. When compared to the gold-standard in-clinic 6MWT, it is accurate, well-correlated, and shows construct validity. Home-based tests may increase variability than in-clinic tests, however, incorporating physiological data from wearable sensors, such as cardiac effort, or aggregating the data could mitigate this and improve interpretability. Despite the substantive evidence around the feasibility of a digital 6MWT, systematic longitudinal data is lacking, and more work is required to demonstrate the ability of the digital 6MWT to reflect disease severity, show sensitivity to change and establish an MCID for it to be implemented into clinical trials and practise. There is an open question whether structured exercise tests hold more value than exercise metrics derived from passively collected data, which is yet largely unexplored. While numerous challenges regarding data collection and processing remain, future clinical trials should move their focus away from initial validation towards assessing the longitudinal stability and sensitivity to change in digital endpoints.

## Acknowledgements

*We would like to acknowledge Isla Kuhn and Clare McQuaid from the University of Cambridge Medical Library for their help with the literature search.*

### Financial support and sponsorship

*None.*

### Conflicts of Interest

*M.T. reports consulting or advisory roles with Janssen Pharmaceuticals, Merck Sharp & Dohme UK Ltd, Apollo Therapeutics, RxPx and uMed Ltd.*

## REFERENCES AND RECOMMENDED READING

Papers of particular interest, published within the annual period of review, have been highlighted as:

▪ of special interest
▪▪ of outstanding interest
